# The Transatlantic Cattle Trade and Its Regulations from a Veterinary Point of View*Read before the United States Veterinary Medical Association, Sept. 16th, 1891.

**Published:** 1891-10

**Authors:** Williamson Bryden


### THE TRANSATLANTIC CATTLE TRADE AND ITS REGULATIONS FROM A VETERINARY POINT OF VIEW.*

By Williamson Bryden, V. S.

Within the last year the American Export Cattle Trade has been before the public more conspicuously than at any time since its commencement, some fifteen years ago.

This has arisen principally from recent legislative and other interference at home and abroad, with a subject, imperfectly understood, not fully developed, and that has been grossly misrepresented. It is consequently regarded by those engaged in important branches of the business as in many ways unfair to them, and an injustice which cannot possibly benefit the animals, or improve materially their freedom from hardships in transportation It is a blow, especially, at the ships, no matter what their class is or what ports they sail from, up a river, 130 miles or within an hour of the open sea.

This traffic, as is well known, suddenly assumed large proportions. At first both lean and fat cattle were shipped, until fear of contagious diseases stopped this and permitted only fat animals, fit for slaughter, in quarantine, on landing, and dressed beef.

At first, as with all new undertakings, a period of immaturity had to be passed. Ships built for other kinds of cargos had to be converted into cattle boats, which was in most cases easily done. These were of different classes and tonnage. The crews may have been at first unused to ships with such cargoes and ventilation on some not as perfect as now. The cattle men were unused to the sea, many of them entirely unfit and often ill paid, so losses occasionnally resulted, that could possibly have been avoided.

During the same period our Inland transportation systems were also imperfect and unprepared for the increased business, the stock yards and resting places poor and inadequate, the cars plain and primitive, many of them old, often overloaded and without sufficient experienced attendants. The animals were consequently neglected. Some died on the journey before reaching the yards, from which the survivors had often to be hurried to the ship without proper rest; bruised, tired, hungry and

---

* Read before the United States Veterinary Medical Association, Sept. 16th, 1891.

thirsty, it is no wonder that some died at sea, and directed every one's attention to the ships, unjustly blaming them as the cause of all these losses.

In spite of all this the business continued to grow, losses not being generally excessive, and insurance rates declined as those engaged in handling the cattle gained experience.

The British stock raisers could not supply their commercial, manufacturing, and working populations with sufficient home raised beef and mutton products, which they must have, at the lowest prices ; and which this and other countries had enough of and to spare, so the business increased and flourished, untrammelled by regulations or restrictions. Its rapid increase, however, made it necessary for the Privy Council of Great Britain to take some action in the matter to satisfy conflicting political, commercial and agricultural interests. For example :—Land owners, to whom the farms belonged, and the tenants who leased them for long periods at high rents, became alarmed at the great and sudden invasion, fearing that it might prove to their disadvantage and perhaps their ruin, even if the experiment was finally successful. Some politicians used the subjects for party ends. Others believed in Protection, or in fair play between nations. While others again believed that the British Colonies ought to get all the benefit of supplying her wants. The friends of Ireland, too saw in such an influx, espcially of lean cattle, a menace to their regular business in supplying English and Scotch farmers, who raise large crops of roots, fodder, etc., with young, lean animals for fattening. Consequently all such influences had to be considered, all such interests had to be dealt with.

When to these were added the fear of contagious diseases, from which the agriculturalists and stock raisers of Great Britain have suffered so much within the last 40 or 50 years, the objections and fears of the farmers, especially, were to be expected.

The discovery soon after this trade opened that Contagious Pleuro-pneumonia was wide spread in some districts of the Eastern States, and its subsequent appearance in the West, the extravagant reports of the prevalence of Tuberculosis made by United States and State officials, the alarming and sensational methods adopted by them to eradicate it, as witnessed in Maine and other parts of the Eastern States, where instead of individuals being condemned, not only whole herds were slaughtered, but animals sold long before, if some relationship conld be traced, were hunted up all over the country and dealt with in the same summary manner. The fact of dealing with such a disease more harshly than they would with Contagious Pleuro-pneumonia, surprised every careful student of the subject and led many to think that our cattle population was in a deplorable condition. At all events, it forced many people abroad to suspect that what was being called Tuberculosis was really a dangerous extention of Contagious Pleuro-pneumonia, and yet this did not exhaust the list of obstacles for still other diseases such as splenic fever, anthrax, actinomycosis, etc., annually during what ought to have been the best months of the year for shipping cattle, demanded their sacrifices.

When all this is considered, the moderately conservative course pursued by the Privy Council of Great Britain would surely seem deserving of applause, although at the same time it makes one wonder what their course will be when the United States is declared free from contagious diseases, which the Bureau of Animal Industry assures us they will be soon able to do, and which we all hope will be the case.

The opportunity is certainly a grand one for them to show practical statesmanship of a high order, and which will remedy in part at least the unfortunate state of things, which took the meat supply out of the hands of the native farmers and stock raisers where it *naturally* belongs. As occurred when fat cattle and dressed beef only, dared be admitted. Surely there can be no great danger in admitting healthy, young "store," as young animals for fattening are called, the farmers would again be "in" the business, and so would British railroads and smaller ships.

Suppose a Country well stocked with two year olds, born and bred in a natural way on the prairies and plains, from improved herds, suckled by their mothers—not on skim milk or slops—should not such animals increase greatly in size and ought to pay well for feeding. They are raised under circumstances, that makes them not only hardy, but that fits them for any kind of a journey. The danger from such to the farm stock of a Country must be trifling indeed. Another important thing to be considered is the liability to sudden interference with supplies from long distances in the event of war, fat cattle and beef would be exhausted in a week, whereas a Country full of young "stores" would last a year—besides improving and fertilizing the farms so that thus crops would double.

It must be the veterinarian's, as it is the scientific solution of the whole business if politic and questionable measures do not interfere. It also offers a solution to the humane side of the question, if carried on under proper restrictions and regulations, applied, not to the ships alone, but to the whole journey whether on land or sea. For if old ships are condemned, why not old cars, cattle yards, etc.? applied, not to mean unscruplous ship owners, but to a reckless, unscruplous class of shippers and foolish Legislators who have caused by far the most trouble.

The great fault with "good" friends like Mr. Plimsoll, earnest well-meaning people no doubt, is, that their methods of investigating the subject have given them false impressions of the share of blame the ships are liable for. Of course, there are good ships and poor ones, good ship owners and mean ones. He has done the regular lines great wrong and injustice. For the fact is that reckless shippers have been largely to blame for the hardships the animals have suffered, being subject to no regulations in handling their cattle, for none worthy of the name have ever been carried out on the Inland half of the journey, and more than half of the help sent to sea with their cattle has been ill paid and worthless.

The movement of large bodies of cattle has always been attended with some degree of hardship from the times when they had to be driven from Market to Market to the present, and half of the lives of most of such animals is made up of hardship; this must not be lost sight of. Those sheltered and cared for—in warm places—while being fattened cannot endure the fatigue and exposure that animals can that have grown hardy and strong on their native plains under scorching heat and droughts of Summer and the storms of Winter. The *class* of animals that ought to be allowed to be shipped is therefore the most important consideration in determining what regulations are necessary; the alterations required for the ships being entirely a secondary matter, and something that can easily be settled after.

Soon after the commencement of this traffic and up to a year ago great improvements were made in the cattle cars which led every one to believe that the animals would be greatly benefited; whether this was due to the energy of the patentees and the Companies controlling them, the enterprise of the railroads, the demands of the shippers, or all combined, I cannot say, at any rate "hope told a flattering tale" for a greater average of comfort to the poor beasts was at least made possible, and if proper regulations should be enforced upon unscrupulous shippers and slack practices, much benefit would follow. But as the greater efficiency of these cars with their conveniences for feeding and watering, improvements in brakes and couplings and other attachments, and contrivances which doubled the rate of speed at which such trains were formerly run, became demonstrated, the example of the willing horse that just gets more to do, repeated itself. For instead of the proper complement of animals being carried, and resting places stopped at, it is now no unusual thing to find two or three extra ones crowded into the car load and hurried through without any attention. Thus, the animals do not receive the benefit of such improvements, as all the abuses incident to the old cars have been repeated. Of course there are shippers who handle only the finest cattle and take proper care of them as well as of any class of animals shipped by them. They are not all reckless. The promised improvements in the stock yards and resting places at our Ports have not been carried out; they ought to belong to the Government, but must not be located as was blunderingly done in the case of the Boston Cattle quarrantine which is a disgrace to the Department. Many of the yards remain the same as at the commencement; one port at least, Portland, has none. At different times during the Spring months, when the frost is leaving the ground and the weather is rainy, it is no unusual thing to find the cattle standing up to their knees — yes to their bellies in mud, the poor creatures being unable to drag themselves through it to lie down; or to reach water and feed, the yards being often in this condition for two or three weeks at a time. Then again in Summer, they are exposed to the burning sun without shelter, their tongues hanging from their mouths, and they panting as though the breath would leave their bodies. To animals that have been fattening in idleness under cover for months, this is a very poor preparation either for the rest of the journey, or the rest such a journey demands, without even counting their sufferings in open sided cars in the storms of Winter.

Nothing could better illustrate the point that it is useless to put high standard restrictions on the ships before the inland systems are on a level with them, than the fact that some of the finest ships ever constructed for the business have within the last six months lost as many cattle as were lost on the smaller ones, which they replaced, during the same length of time. I allude to the Leyland Line. This was from no fault of the new ships. The shippers sent a class of animals unfit for shipment, when forced to undergo hardships from reckless treatment and abuse before reaching the dock, a fact which the United States officials are perfectly cognizant of, and still overlook.

Mr. Leyland, who owns one of the finest Lines that ever crossed the Atlantic, said at the recent investigation in London:—"it is not a question of stopping the trade, but of regulating it,"—or words to that effect, and it is the key to the whole subject, to look out for reckless ship owners and shippers and slack practices every where.

As is well understood, this matter must be regulated, but the ships must not be made the only sufferers. Neither for that matter must the Inland systems, or any other. There is no need for any regulations that will either destroy or embarrass the trade, or that cannot be easily arranged and enforced. Every branch of the business must be in harmony with the other, and the great nations engaged in it must be just to each other, and see to it that all regulations and restrictions are framed and impartially carried out; not through the tricks and bounce and bluff that scheming traders resort to, but as would be expected between two great honorable nations. No order even from the highest sources must be allowed to pass diseased animals when justly and wisely condemned, as was recently done in Boston with scale sheep.

In some respects the recent investigation in London may result in good in the future, in others it has already done great harm, and in one instance its effect has even been *unpatriotic*, as when the United States is encouraged to deal with British ships in the harsh arbitrary way they are doing. For proof of this, I will take the liberty to quote from a letter to me from the department at Washington, when appealing to them for a delay of the 2 feet 8 inches clause in the cases of Lancastrian and Philadelphian which says of the regulations that they "meet the objections raised by English authorities, and will most probably prevent their issuing such stringent measures as might have driven all vessels out of the transatlantic cattle trade." What nonsense! A party that would encourrage such a thing would not last a year in Great Britain.

These regulations have consequently been carried to such a high standard that ships built expressly for this trade by the highest authorities in marine architecture do not reach it. Not only this, but the enforcement of the restrictions imposed are being carried out so long before the regulations affecting the Inland systems of transportation, etc., can be carried out, that it is a piece of patchwork and not creditable to the fairness that ought to govern such transactions between nations. The improvement of the stock yards, by building shelters and drainage, the class of animals fit for shipment, their size and weight, the number allowed in a car, etc., these things must have been wilfully lost sight of altogether. The number that die on the Inland journey to Port is not known, and post mortems are seldom or never made, disinfection is hardly thought of *excepting on the ships*, all this part being a "go as you please."

This solicitude for the condition of British ships is unbounded, even to the extent of inconsistency. Heavier fittings are demanded, although their fastening to the deck is the same, stantions are substituted by heavier ones, although they could have been strengthened with wood, which has always proved sufficient and only makes extra expense ; spaces are widened, instead of regulating the size and weight of the cattle, if 2 feet 6 inches is enough on the spar deck it must be enough on the main deck ; valuable space is sacrificed for alley ways, instead of employing an extra man or two, not "stiffs," but men paid for this work, which only keeps them busy about ten days in a month, the other twenty days of their engagement being spent in idleness. All this power is in the hands of the Department, yet they have not used it. The bill confering this power passed without Boston's members of Congress knowing that their port, the finest in the world, was being classed with Philadelphia 130 miles up a river, their steamship agents too simply bowed their heads and accepted the situation, poor creatures.

No finer opportunity was ever offered the veterinarians of any Department to distinguish themselves by a splendid philosophic interpretation of this vast subject, no worse failure to grasp the situation with professional comprehensiveness and justice could well be presented; the assumption of the role of shipwrights and marine architects by them and the neglect of proper professional work is a subject they must expect to be criticized for. Were the case reversed, and United States ships subjected to such treatment by a foreign country under similar circumstances, I am very sure it would not escape Mr. Blaine's attention, or be allowed, without a vigorous protest and demand for impartial treatment; every concession demanded from his ships having to be met by equal improvements on the first stages of the journey; well might another committee be appointed to investigate this end of the business.

